# Digital technology adaptation and initiatives: a systematic review of teaching and learning during COVID-19

**DOI:** 10.1007/s12528-023-09376-z

**Published:** 2023-04-26

**Authors:** Xue Zhou, Christopher James MacBride Smith, Hosam Al-Samarraie

**Affiliations:** 1 School of Business and Management, Queen Mary University London, Mile End Rd, Bethnal Green, London, E1 4NS UK; 2 Institute for University to Business Education, Glasgow Caledonian University, Cowcaddens Rd, Glasgow, G4 0BA UK; 3 School of Design, University of Leeds, Woodhouse, Leeds, LS2 9JT UK

**Keywords:** Digital technology adaptation, COVID-19, Higher education, Student and staff experience, Systematic literature review, Online/blended learning

## Abstract

COVID-19 dramatically influenced students’ and staff’s learning and teaching experiences and approaches to learning. While many papers examined individual experiences in the context of higher education, synthesising these papers to determine enabling and hindering influences of digital adaptation was needed to guide the next phase of online learning reforms. This study explored the main dimensions of digital technology adaptation in higher education during the COVID-19 pandemic. The consequences for student and staff experiences and what aspects should be sustained and developed were discussed in this review. A total of 90 articles (published between 1st January 2020 and 30th June 2021) were identified and analysed based on the preferred reporting items for systematic reviews and meta-analyses framework. Four dimensions (with associated sub-factors) were found to influence student and staff experiences: techno-economic; personal and psychological; teaching, learning and assessment; and social. The findings highlighted that an integrated approach, across institutional, technical platforms, and individuals would be required to sustain digital learning initiatives during the crisis time.

**Supplementary Information:**

The online version contains supplementary material available at 10.1007/s12528-023-09376-z.

## Introduction

COVID-19 has had a significant impact, whether this has been dealing with bereavement, ill health, or coping with government public health controls and levels of lockdown. While many areas of life were suspended, governments around the world have been in a constant search for ways to keep education going (Müller et al., 2021). Universities, their staff and students were forced initially into ‘Emergency Remote Learning’ and then continued with online learning or adjusted to a hybrid approach of on- and off-campus delivery. Consequently, there has been a significant adoption of and adaptation of various digital solutions to support online teaching and learning effectively; adoption and adaptation were recognised as two sequential stages in deployment (Venkatesh & Bala, 2008). Technology adoption models, such as Technology adoption model 3(TAM3) (Venkatesh & Bala, 2008) and unified theory of acceptance of use of technology (UTAUT) (Venkatesh et al., 2016) have validated general factors that influence the use of technology, including psychological, social, facilitating conditions, and system. Moreover, Venkatesh et al. (2016) developed a multi-level framework for UTAUT, where higher level attributes, such as (physical) environmental, geographical location and organisational, influenced technology acceptance and usage. Bala and Venkatesh (2016)’s adaptation model indicated influencing factors such as experience, training effectiveness, psychological engagement, management support, and reaction to change based on threat or opportunity determination; responses were mediated by either emotional or environment-influencing strategies. In a similar vein, personal awareness, ability, organisational management, and collaboration among the colleagues also been found to impact on the academic staff technology adoption by using concerns-based adoption model (CBAM) (Petherbridge, 2007). In recognition that published studies of staff and student experiences detailed adoption of and adaptation of technology due to COVID-19, but did not consistently indicate their use of technology pre-COVID-19, then the term adaptation (as later stage) has been utilised in this research to encompass both adoption and adaptation. Consequently, between the models of technology adoption and acceptance, influencing factors were generalised initially for this research as technology/systems, social, and psychological, including the need to consider higher-order attributes (beyond the individual); these general factors and attributes were used in coding (see methodology section).

Most studies on digital technology adaptations during COVID-19 provided programme, course and institutional level cases that presented a varied situation of acceptance, adaptation, and desire to continue with a blended approach or return to face-to-face learning (Ghazi-Saidi et al., 2020; Hattar et al., 2021; Sebbani et al., 2021). Holistically, these cases were contradictory around experiences of staff and students and do not provide a synthesis of available international empirical cases to examine commonalities across domains (institutions, disciplines, and technologies). The few systematic literature reviews (based on articles published around adaptation in Higher Education in first few months of COVID-19) highlighted that pragmatic approaches by academic and technical teams were taken, which resulted in mixed opinions of quality, efficacy, and efficiency. Such rapid adaptation was met with resistance from some staff and students, in part due to unproductive study spaces and mental wellbeing issues. Additionally, technology challenges created issues in quality of, and access to teaching and learning resources, including in laboratory and practice-based activities (Deng et al., 2021; Maddumapatabandi & Gamage, 2020; Mseleku, 2020). Positively, the changes in response to COVID-19 have driven innovation and exposed staff and students to new forms of teaching, learning and assessment that have potential to improve experience, yet further research was advocated (Talib et al., 2021). This systematic review provided an updated, integrated perspective around institutional, technology and individual level adaptations and experiences that would inform future practice. In the context of technology adaptation during COVID-19 (an enforced step-change in practice), then it was important to examine what specific dimensions influenced experiences (coping) across a range of disciplines. Based on these observations, this study aimed at answering two research questions: (1) What are the main dimensions affecting students’ and staff’s learning and teaching experiences during COVID-19? and (2) What are the initiatives required to enhance digital technology adaptation in the post-COVID-19 period? The aim of this systematic review was to explore digital technology adaptation in higher education and its related impact on the experiences of staff and students during the COVID-19 pandemic, and how to use the experiences to form a supporting HE environment to improve the digital technology adaption.

## Methodology

The Preferred Reporting Items for Systematic Reviews and Meta-Analyses (PRISMA) guidelines by Liberati et al. (2009) was applied to guide the review and answer the research questions outlined earlier.

### Literature search

This review consisted of previous empirical research on students’ and staffs’ learning and teaching experiences during the COVID-19 pandemic. The search of previous studies was based on predefined database sources: ERIC, Education Database (Proquest), and SCOPUS. Most of the titles identified outside the primary search were not detected by the Web of Science database because they did not contain the set of search terms that we used in this study. Thus, this review used the SCOPUS database because it contains both the ISI and Scopus indexed rank papers (Oakleaf, 2009). Google Scholar was also used because some of the publications were not published in scientific journals and their impact cannot be tracked by scientific citation (Ficko et al., 2019). Empirical studies (e.g., qualitative, quantitative, and mixed methods) were included that were carried out across different, global university settings. All relevant empirical studies published in peer-reviewed journals, and conference proceedings (for which full-text was available to researchers) were included in this review.

In addition, only English-written articles that were published between January 1st 2020 and June 30th 2021 were retrieved and processed in this review. This start date was chosen because it demarcates the spread of COVID-19 across the globe (in accordance with the declaration of World Health Organization). Search records were retrieved on 12th July 2021. The following keywords were used in the search: (“Covid” OR “Covid-19” OR “pandemic” OR “post Covid”) AND ("digital learning" OR “online learning” OR “blended learning”) AND (“higher education” OR “university” OR “tertiary education”) AND ("interview" OR "questionnaire" OR "survey" OR "focus group" OR "case study") AND ("students" OR "learners" OR "tutor" OR "Faculty" OR "lecturer" OR "teacher") AND "experience". Boolean operators and quotation marks were utilized in the search to identify potential intersection between the keywords and facilitate the retrieval process of variations found in the lexicon related to students’ and staffs’ learning and teaching experiences.

### Screening and coding procedures

**Fig. 1:**
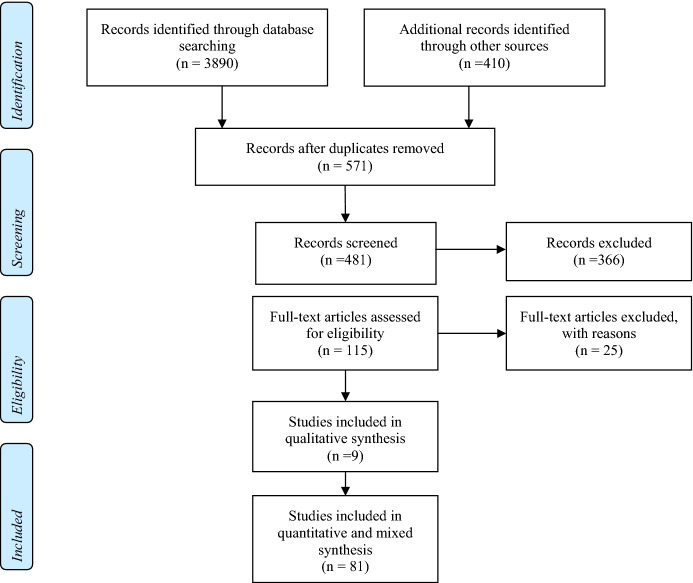
Article searching and selection process based on the PRISMA statement

The initial search result of the literature was 4300 empirical studies (including additional records from cited works). An initial screening of titles and abstracts of these studies was conducted to determine the relevance and value of each study to the current review. Articles were included based on certain inclusion criteria: publication (the article was available in a peer-reviewed, scholarly journal or conference proceeding); language (the article was written in English); content (the article investigated the experiences of students or staff during COVID-19); and context (the sample in the retrieved articles was limited to university students and staff). After applying these criteria on the retrieved articles and excluding of duplicates, 571 studies remained. Other systematic, scoping, and conceptual papers (e.g., ideas and opinions) related to university students’ learning and non-peer-reviewed research were also excluded; there were limited scoping and systematic reviews, and these were used to inform research questions in this study. A total of 481 studies were excluded during the screening of the full article because some studies did not clearly report the impact of COVID-19 on students’ learning experience, or methodologically had small sample sizes (< 50 for quantitative studies to ensure sufficiently representative of capturing representative experiences (Kelley et al., 2003)) or too few for trustworthiness in qualitative studies (Boddy, 2016; Korstjens & Moser, 2018). As such, 90 articles remained in the final phase (see Fig. 1).

A literature matrix was created and used to help in the review process of the identified articles. The matrix consisted of multiple columns (study features) and rows (studies). Over and above bibliographic information (citation, article title and journal) in each row, eight study feature groupings were coded for each study in this review: (1) country of study, (2) discipline area(s), (3) methodological characteristics (study design, type, and sample size), (4) technology characteristics, (5) factors, (6) benefits, (7) challenges and (8) recommendations and implications. The first two features were important to determine regional and discipline coverage of included studies. The third grouping ensured the article met the inclusion criteria, whilst the fourth captured details of technology types (where specified). The features in the fifth grouping along with the sixth and seventh groupings recorded how digital innovations influenced learning and teaching (so related to the first research question), with initiatives relative to the second research question captured in the eighth group.

The identified articles were read carefully and evaluated by the first and second authors. These authors independently inductively coded the articles using the general factors identified above—technology/systems, social and psychological—for initial framing. A meeting between first and second authors was set up to compare codes and an iterative process of discussion agreed the naming and content of the codes (sub-factors) relating to students’ and staff’s learning and teaching experiences. Through this coding process additional dimensions (themes) and sub-factors were agreed. As a result, four main dimensions emerged: techno-economic; personal and psychological; teaching, learning and assessment; and social. Intercoder reliability was used to indicate the intercoder agreement for article classification across the authors. The obtained Krippendorff’s alpha for the four themes were above the recommended level of α = 0.8. These dimensions were interpretable in accordance with the technology used by students and staff across different disciplines. These dimensions were used to code students’ and staff’s learning and teaching experiences during COVID-19, as an item-focused coding approach was effective in outlining relevant themes across university disciplines.

### Quality check

Three reviewers evaluated independently the final list of articles. The following criteria were used to assess the quality of each article:

1. Relevance of the study objectives in addressing the research questions.
2. Appropriateness of the study design for the stated purpose.
3. Appropriateness of the study type and relevance to the focus of the review (empirical quantitative, qualitative or mixed methods).
4. Reliability of the results in relation to the focus of this review (trusthworthiness of findings for qualitative findings; reliability/generalisability of findings for quantitative results).

The interrater reliability value was calculated using an item-by-item method, specifically by dividing the tally of agreements by the total number of agreements and disagreements, divided by 100 (Cooper et al., 2007). The average value for the interrater agreement was 89%.

## Results

### Studies’ characteristics

**Fig. 2:**
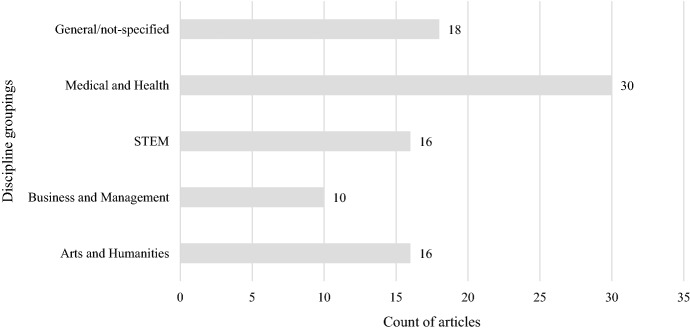
Disciplines covered in studies

Of the 90 included articles, 63 studies (70%) of the reviewed studies were focused on students’ experiences whereas 12% examined staff experiences and 18% considered both staff and student experiences. Figure 2 shows that 30 studies (32%) were conducted in the Medical and Health discipline, and 16 studies (18%) from Science, Technology, Engineering and Mathematics (STEM), 16 studies (11%) in Business and Management, 16 studies (18%) Arts and Humanities, and 18 studies (20%) that did not clearly specify discipline. Additionally, 65 studies (72%) used a survey method, 16 studies (18%) used interviews, 7 studies (8%) used focus groups, and 2 studies (2%) used mixed methods.

A total of 87 studies (97%) examined adaptations to teaching and learning, 23 studies (26%) reported to adaptations to assessment and 2 (2%) around wider support in the response to COVID-19. A range of digital technologies were used and adapted—across synchronous and asynchronous teaching and learning, as well as in assessment (Table 1). Some studies focused on particular technologies and adaptations (Nel & Marais, 2021), but more commonly research was focused on the overall experiences of adaptation to online learning. The clarity of the level of acceptance and use of specific technologies pre-COVID-19 at each institution was provided in only a few studies, consequently making it challenging to determine the pre- and post-experiential changes in experience in response to adaptation of technology use.

**Table 1:** Examples of technologies mentioned in selected studies

Types of technology	Examples of apps
Communication	Zoom, Microsoft Team, QQ group, WhatsApp, WeChat, Skype, Google meet, Cisco WebEx conference, Tencent conference, GroupMe
Learning management system	Canvas, MOOC, Tencent courses, Blackboard, Moodle, Google classroom
Social media apps	YouTube, Virtual whiteboard, Stud.IP, Facebook group, LinkedIn
Document management tool	Google Suite (e.g., Google drive), Net.Create
Online curriculum provision	Simulation, vSimGPclinics, ebooks
Engagement tool	Doodle Poll, Qualtrics, Padlet, Online discussion forum
Assessment	E-test, online self-test, Exam integrity tools (Lockdown Brower), Exam proctoring tools (e.g., Respondus webcam, Monitor), e-learning Portal, ProctorU; Publisher online testing

### Digital technology adaptation during COVID-19

**Fig. 3:**
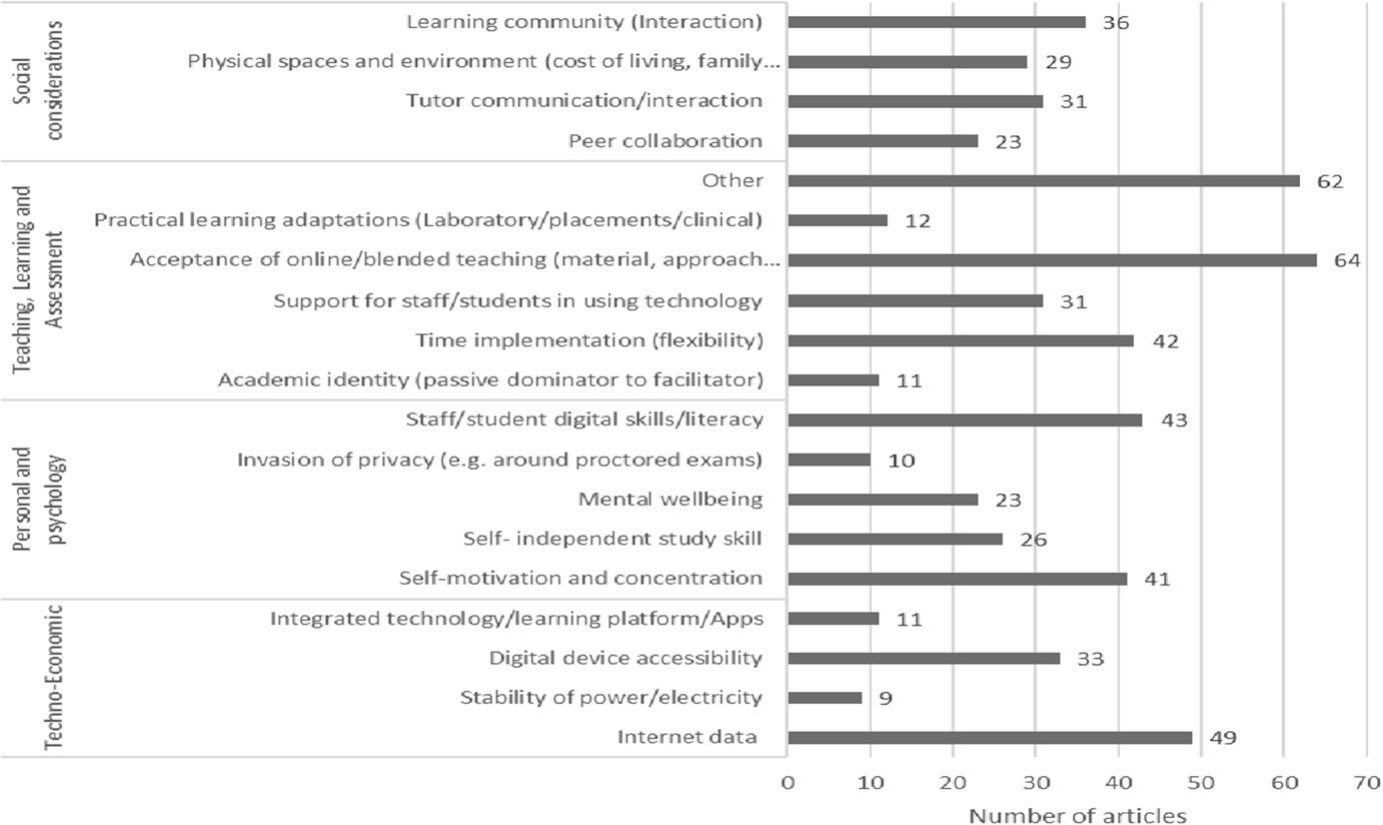
Dimensions and sub-factors affecting digital technology adaptation during COVID-19

Our review of the 90 articles revealed several characteristics common among students and staff in relation to adapting digital solutions during the COVID-19 pandemic. We categorized the identified sub-factors into four dimensions: Techno-Economic; Personal and Psychology; Teaching, Learning and Assessment Practice; and Social (see Table I in the supplementary file). Figure 3 outlines the dimensions, their sub-factors and article count.

#### Techno-economic

The Techno-Economic dimension encompassed a number of considerations: (1) infrastructure (macro factors that relate to utilities, such as internet and electricity), (2) the platforms and applications used as part of teaching, learning and assessment, as well as (3) more individual factors (such as access to hardware and affordability of access).

This review revealed that access to the internet was as an important influencing factor for both students and staff (N = 49, 54%)—with those with faster and more stable access having a more positive experience. Moreover, the level of access was not typified by country, with studies indicating both sufficient access and access frustrations in the same country; for example, in Germany—acceptance: (Schlenz et al., 2020) and frustrations: (Eberle & Hobrecht, 2021) were evident. Furthermore, the impact of contrasting levels of ease and quality of access between rural and urban areas was indicated by some studies—Calder et al. (2021) in New Zealand and Gautam and Gautam (2021) in Nepal. Additionally, sharing of connection in study places impacted on the quality of access (Costado Dios & Piñero Charlo, 2021). It was evident that those countries the existence of clear national policies around online learning (Müller et al., 2021) influenced the readiness to transition to online learning. Overall, these bandwidth considerations created an online environment typified by a lack of connection (Blackley et al., 2021). Moreover, such stability of access led to student frustrations, feelings of isolation and demotivation about their studies, and potentially to their academic achievement.

Equally, the cost of increased internet usage was highlighted as a barrier and strain on families’ finances, even in countries where the network providers offered a free data bundle for educational sites it was not sufficient (Ogbonnaya et al., 2020). This review showed that adaptations made in these more challenging situations saw a move to asynchronous (rather than synchronous) delivery to remove some of these anxieties (Ghazi-Saidi et al., 2020), but in the Emergency Remote Teaching phase then this was not always possible.

Additionally, online learning requires greater consumption of electricity and in some studies (N = 9) and countries this was an issue—whether this was that electricity network had capacity issues (e.g., in South Africa and load-shedding) or whether unstable (Bordoloi et al., 2021; Laher et al., 2021). However, even in some countries with unstable supply, students did not see this as a significant contributing factor (Ogbonnaya et al., 2020), perhaps reflecting that they had already developed strategies to manage such issues.

In terms of use of platforms and applications, the results also showed that enhanced efficiency in handling assignments for staff (Chen et al., 2021), as well as increased use of online formative and summative tests were evident (Laher et al., 2021). The use of different forms of content and complementary apps was evident through the use of tools such as Net.Create and Perusall (Craig et al., 2020; Nel & Marais, 2021). However, a proliferation of different apps and lack of consistency between different lecturers and courses created challenges for the student experience (Ghazi-Saidi et al., 2020; Oliveira et al., 2021). Moreover, platforms became unstable due to heavy use, and financial implications for students to access different apps impacted on their teaching and learning experience. Some of these platform challenges were related to Emergency Response Teaching early in the pandemic.

At a more individual level, then access to appropriate devices was required, ranging from smartphones to tablets and to laptops and computers. The level of access to such a device was related to each student’s socio-economic status and influenced their level of satisfaction (Al-Salman & Haider, 2021). Some universities provided discounted hardware and software to their students (Ghazi-Saidi et al., 2020), but this was not always possible. There were many challenges, particularly in pivoting online, that included i) hardware that was not suitable for online learning, ii) that apps were not compatible with smartphones (that were used heavily), iii) difficulty in maintaining devices (due to lockdown) as well as iv) financial constraints on buying a new device (when incomes had dropped). Such challenges had the potential to exclude students due to a lack of suitable devices, which in turn created anxieties for students about their studies and about graduating.

#### Personal and psychological

This dimension referred to the personal skills (motivation and independent learning skills), and psychological status (mental health) to cope with online learning. Several studies (N = 41, 46%) demonstrated that students were likely to experience some difficulties in managing their own study (Abou-Khalil et al., 2021; Hijazi & Alnatour, 2021; Rizvi & Nabi, 2021; Ruiz et al., 2021). Weak motivation for engaging with online learning and lack of concentration were found to be the significant issues for students adapting to online learning (Al-Tarawneh et al., 2021; Lassoued et al., 2020). Students were also distracted by their family responsibilities, (e.g., home schooling and caring) and a physically uncomfortable and chaotic home environment (Al-Rasheed, 2021; Gonçalves et al., 2020). These experiences were also shared by the members of staff, whose productivity was reduced due to the difficulties in concentrating on remote classes and multi-tasking. In addition, some studies (N = 26, 29%) also explored to what extent the students’ learning effectiveness had been affected by the independent study skills, such as self-regulated learning (Ogbonnaya et al., 2020), problem-solving skills (Eberle & Hobrecht, 2021), self-efficacy (Martha et al., 2021), and time management skill (Almazova et al., 2020; Ogbonnaya et al., 2020). Students with better independent study skills were more active in approaching tutors for support and, as a result, enjoyed the online teaching more (Müller et al., 2021).

Additionally, 26% of the studies highlighted the importance of considering mental wellbeing. Online learning reduced interaction between peers and tutors which heightened anxieties and stress, even though chat and communication were still possible for students and staff through various channels. Students reported that they felt stressed and anxious for their academic achievement, employability, health, and work-life balance (Secundo et al., 2021; Sugino, 2021; Watermeyer et al., 2021). Students at master's level experienced more angst than those at undergraduate level, as they took more responsibility for their financial arrangement and were worried the pandemic would delay the process of rebuilding their formal career (Gautam & Gautam, 2021). In addition, students pointed to their discomfort with the proctored online exam and the intimidating monitoring systems (Chen et al., 2021) that universities had introduced to maintain academic integrity of assessments, particularly examinations. Such systems heightened students’ stress levels, as it might over-stretch the availability of the internet data and violate students’ privacy (Morgan et al., 2021).

The findings (N = 43, 48%) showed that students and staff’s digital skills—the ability to work with various apps, software, and search for e-resources contents—improved significantly since the onset of adapted teaching and learning approaches (Händel et al., 2020; Khalaf et al., 2020). A notable number of students and staff demonstrated their confidence in using digital devices, learning platforms and apps essential for their teaching and learning, especially in those universities that provided sufficient training and support (Casacchia et al., 2021; Ghazi-Saidi et al., 2020; Stewart & Lowenthal, 2021). However, some students were less prepared to use various technologies in the sudden transition (Al-Rasheed, 2021; Colfer et al., 2021), and more staff over 55 years old also required extra instruction and IT support (Almazova et al., 2020).

#### Teaching, learning, and assessment

This dimension related to the changes to staff’s self-concept when engaged in online learning, as well as the views and outcomes of support in moving online. This dimension also involved the acceptance of online learning and specific technology adaptations in particular discipline areas. Several studies (N = 11, 12%) examined the staff’s motivation and any perceived adaptations to their academic identity. Whilst for some this represented a new ‘intellectual challenge’ and an opportunity to master a different form of teaching, (Almazova et al., 2020; Müller et al., 2021; Secundo et al., 2021), other studies felt that the move challenged the ‘core academic values’ and purpose of a university and inhibited ‘holistic learning’ (Müller et al., 2021). Moreover, some staff felt that they had become more like technicians and that teaching and learning have become passive (Hijazi & Alnatour, 2021; Müller et al., 2021; Watermeyer et al., 2021).

The flexibility of online learning was strongly identified (N = 42, 47%) with time and cost-savings being outlined (e.g., lack of commuting), more strongly for working students (Casacchia et al., 2021; Costado Dios & Piñero Charlo, 2021; Gautam & Gautam, 2021). Moreover, online meant that the schedule was no longer dictated by physical spaces on campus, so allowing flexibility with the endpoint of sessions (Toader et al., 2021). Additionally, the options to self-pace were both appreciated (Istenič, 2021; Kasai et al., 2021) and not appreciated (Laher et al., 2021). In particular, the ability to replay recorded videos was identified as allowing students the opportunity to master concepts by re-listening and being able to control the speed of playback. However, the resultant balancing of personal and academic time was impacted—with lecturers spending more time on preparing materials and students indicating increased workloads and distractions to their learning at home (Al-Rasheed, 2021; Oliveira et al., 2021; Stewart et al., 2021).

Support for staff and students (N = 31, 34%) was another factor, with positive institutional support being associated with a more positive perception of online learning (Al-Karaki et al., 2021; Mouchantaf, 2020). This support came in the form of (a)synchronous training, technical support, and peer support (building stronger connections between academic colleagues through necessity). Conversely a lack of support for staff resulted in a perception of teaching preparation taking too long, thereby creating negative views and barriers to acceptance of online learning (Hayat et al., 2021; Lassoued et al., 2020). Studies identified the greater pastoral role the academic staff had undertaken (Watermeyer et al., 2021), in part due to lack of access to student support services (Istenič, 2021), which increased staff workload further. Going forward, staff and students need to be supported in how to work in a digital work and learning environment, and doing so would equip both groups with relevant transversal skills (Almazova et al., 2020).

Approaches and adaptations associated with clinical and practical work indicated innovative adaptations—in the use simulated electronic health records (Kasai et al., 2021), simulated GP clinics (Rasalam & Bandaranaike, 2020) and VR simulations (De Ponti et al., 2020)—but not all disciplines were able to find suitable alternatives. Several papers viewed online learning as being appropriate for the theoretical and written practice elements of a programme, but did not believe that they could replace preparation for practical aspects, such as clinical work, medical interviews, laboratory practice, engineering design, and laboratories (Ahmed et al., 2020). These studies reflected discipline differences around acceptance of online learning, with Arts and Humanities having a higher acceptance of online learning in the future, and Sciences, Engineering, Health, Medicine and Languages expressed concerns and barriers about completely online education (Al-Salman & Haider, 2021). A mixed mode of online and face-to-face emerged from these studies (with more acceptance of online for theoretical aspects), and opportunities to innovate further with the use of simulation, VR, and other tools. Such modality would align with active forms of instruction that are known to be more engaging.

The acceptance and adaptation to online learning was influenced by the level of study, as studies highlighted students in higher levels of undergraduate and in postgraduate study coped better with the move online, whereas level one and two students found the transition more difficult (Khalil et al., 2020; Klein et al., 2021; Yu, 2021). Such differences potentially reflected a lack of familiarity with systems and culture of the university of newer students, as well as readiness to engage in online instruction.

Adaptations to assessments were identified in 23 papers. Students liked unproctored, open-book examinations that encouraged deeper thinking and considered real-world applications (Chen et al., 2021; Morgan et al., 2021). Studies recognised the importance of academic integrity (and the need for proctored examinations) thereby creating a conundrum that will need to be addressed to ensure fair outcomes (Müller et al., 2021; Reedy et al., 2021). In terms of online examinations, particular difficulties were evident in the sciences (Al-Salman & Haider, 2021; Elfirdoussi et al., 2020; Reedy et al., 2021) and academics were not convinced by the integrity of online assessments (Al-Karaki et al., 2021).

#### Social

This dimension consisted of four sub factors, which are (1) peer collaboration (N = 23, 26%), (2) Tutor Communication and interaction (N = 31, 34%), (3) Physical space and environment (N = 29, 32%), and (4) Learning community interaction (N = 36, 40%). Firstly, students acknowledged that staff’s availability to take students’ questions expanded through the adoption of multi-communication channels in online learning environment (Brooks et al. 2021; Müller et al., 2021; Stewart & Lowenthal, 2021), and they could receive a rapid response to their inquiries from their tutor (Al-Balas et al., 2020). Student-staff interaction has been enhanced in the synchronised classes, academic staff adopted different teaching techniques such as feedback, online activities apps (e.g., Qualtrics) to enable students to follow the learning easily (Lambert & Rennie, 2021). Moreover, students can benefit from these initiatives and gain more opportunities to communicate with their tutors confidently, especially those students who were very shy in the traditional face to face learning environment (Müller et al., 2021). On the contrary, some papers highlighted the difficulties in interacting with students online, including inability to check students’ understanding when their cameras are turned off (Toader et al., 2021), unwillingness to engage with online chat function due to the anxiety of being judged by peers (Blackley et al., 2021), and limited time to interact due to the substantial number of participants in synchronized class (Ahmed et al., 2020; Johnson et al., 2020). Findings also demonstrated that some of the staff did not adjust their teaching style and were unable to multitask to answer the questions in the chat during the online teaching (Khairi et al., 2021), consequently, demotivating students to interact with teachers.

Online learning allows students to choose the place where they would like to conduct their study. Benefits include time- and cost-savings (as mentioned already) and comfort of their chosen learning space (Abou-Khalil et al., 2021; Gautam & Gautam, 2021), and spending more time with their families, who acted as important part of the coping mechanism to ensure their mental wellness (Louis et al., 2021). However, several studies also disclosed the limited and chaotic learning space that hinder students’ motivation and their mental health, especially for those students who lived in small apartments and far away from their families (Gonçalves et al., 2020; Martha et al., 2021; Tavitiyaman et al., 2021). Constant disruption caused by the family dynamics, unstable in-house internet connection, increased chores at home made it extremely difficult for students to concentrate on their study (Gautam & Gautam, 2021; Ogbonnaya et al., 2020; Rizvi & Nabi, 2021).

Finally, studies revealed that students desired to connect and bond with their classmates, as the sense of learning community and solidarity among them can enhance motivation to actively engage in the online activities (Almazova et al., 2020; Chen et al., 2021; Frolova et al., 2021; Sugino, 2021). However, twenty-three papers reported that students failed to interact with their peers (Fatani, 2020; Gautam & Gautam, 2021; Händel et al., 2020; Hayat et al., 2021), due to the difficulties in agreeing on the meeting time for group work (Al-Rasheed, 2021), and uneven contributions among the team members (Lambert & Rennie, 2021).

### Potential initiatives in the post-COVID 19 period

This study found that 28 articles (31%) had specific and clear considerations and recommendations, which were used (along with implications of key factors from the first research question) to identify key initiatives. These initiatives spanned infrastructure (national and institutional) and available resources, to institution level actions and finally around creating a supportive learning environment with engaged learners. A holistic consideration is presented here, as dimensions and sub-factors interact with each other.

Firstly, infrastructure aspects (whether national or institutional) were identified as influencing experiences of online learning above. Recommendations from studies indicated government-level responses—investment in Intranet and platform infrastructure (Ahmed et al., 2020; Al-Salman & Haider, 2021) and reduced costs of hardware (Şenol et al., 2021). Cross-institutional collaboration in developing approaches to online education (Lassoued et al., 2020), the creation and curation of Open Educational Resources (OERs) at national level (Bordoloi et al., 2021) to facilitate shared and public courses, and greater use of Open-Source digital learning apps (Elfirdoussi et al., 2020) were proposed.

In terms of institutional considerations, a mixture of off- and on-campus learning envisaged in the future would offer greater options to improve programme timetables, as fewer restrictions on availability of physical space would exist (Gautam & Gautam, 2021; Hijazi & Alnatour, 2021). Physical spaces could still to be used for specific purposes (around clinical, laboratory and development of inter-personal and psychomotor competences) and developing connections, but universities could consider the opportunity presented with any shift to blended learning. Greater provision of IT laboratories (Lassoued et al., 2020) and ebooks (Al-Rasheed, 2021; Bordoloi et al., 2021) is recommended, to address device access issues as well as affording social and study spaces. In the absence of gaining on-campus access, then universities need to consider affordable access to hardware and software (Calder et al., 2021; Majda et al., 2021), and potentially subsidised hardware and software (Ghazi-Saidi et al., 2020).

Enabling social connections should be supported by online learning, for example through break-out rooms (Calder et al., 2021), collaborative digital tools (Craig et al., 2020; Nel & Marais, 2021), and doing so in a creative manner (Rizvi & Nabi, 2021). Collaborative group projects (Ariza et al., 2020) have such potential, but staff must plan on group formation in team or zoom (Craig et al., 2020) reflecting these are new, shared learning spaces. Such new spaces have the potential to be more shared (staff and students) and to be more inclusive of the diversity of learners, such as more introverted learners (Yu, 2021). To achieve this, universities may consider adopting innovative pedagogy, collaborative, and engaging technology-enabled learning spaces (Chen et al., 2021). Such considerations will best inform what can and should be done synchronously and asynchronously (Martha et al., 2021), how to adapt technologies to sustain effective discipline pedagogical practices (Busto et al., 2021), how to develop essential computer-aided skills (Ayadat et al., 2021), how to support practical and competency-based education, and how to create authentic assessments that maintain the academic integrity of results (Reedy et al., 2021).

It is important that staff have been equipped with good level of digital ability to adapt the educational technologies in their teaching (Calder et al., 2021). Therefore, institutional policies and practices will need to be reviewed and updated to ensure sufficient staff digital skills development opportunity is provided. There will be also a need for specialised support, such as Instructional Designers (Johnson et al., 2020; Lassoued et al., 2020), along with ongoing training and support for staff to support adaptation and to help staff see technologies as opportunities to enhance the learning experience and their professional practice.

Equally for students, support is a key initiative that must be taken forward. Developing self-regulated behaviours (SRBs), such as independent study, autonomy, and time management skills (Gonçalves et al., 2020; Montano, 2021), enhancing resilience and self-efficacy, as well as in digital capabilities will be required (Mok et al., 2021). A mixture of pedagogically-aligned synchronous and asynchronous learning opportunities provides an opportunity for these SRBs to grow, as the right mix will balance self-management with scaffolded learning. Adjustment of this balance across distinct levels (or years) of study to support transition and competency enhancement is vitally important. In terms of digital capabilities, the embedding of technology courses (Şenol et al., 2021) and use of technology throughout the curricula, as well as training (Rizvi & Nabi, 2021), has the potential to build confidence and a more positive perspective around technology-enabled learning. Providing these opportunities to recognise and develop the required meta- and transversal-skills will also underpin a better sense of mental wellbeing and of being in control, which will prevent students from becoming anxious; students then have the potential to develop more positive adaptation strategies with enhanced longer-term outcomes. Moreover, these are important skills in the contemporary workplace.

Innovative adaptations have emerged, some of which have ongoing potential and benefits: use of VR and simulation for medical and engineering courses and in Work-Integrated Learning (Almohammed et al., 2021; De Ponti et al., 2020; Iipinge et al., 2020; Rasalam & Bandaranaike, 2020); hybrid teaching (in-class and online) (Busto et al., 2021); online assessments (Al-Karaki et al., 2021; Oliveira et al., 2021; Reedy et al., 2021), as well as those mentioned already above. These innovations have offered new ways of achieving positive outcomes (opportunity of mastery through re-play, more time for discussion, efficiency in assignment management, safe environments to practice and learn). However, care must be taken to ensure that adoption and adaptation of any technology are consistent with creating a positive learning environment and do not create unnecessary anxieties and exclude students (Chen et al., 2021).

Finally, the pivot to online has also brought to the fore, the key issue of accessibility—access to devices, access to internet, accessibility of provided learning materials and accessibility to fair assessments. At an institutional level, the application of universal design principles and practice (Ghazi-Saidi et al., 2020) to create accessible resources (Tavitiyaman et al., 2021) has the potential to impact positively on all students and to unleash the potential of online learning to provide universal availability of learning. To do so, universities must consider the socio-economic and geographically related circumstances of their students and staff.

## Limitations and future works

Both sets of findings supported a multi-level conceptualisation of adaptation (Venkatesh et al., 2016) and demonstrated the impact of macro factors, such as location (associated national infrastructure, policy, and socio-economic conditions), and meso factors such as institutional context (e.g., management support; training availability and efficacy; experience of digital learning technologies), and physical/social environments (e.g., learning and teaching location). These macro and meso-level factors influenced individual factors (such as prior experience of using digital technologies, digital literacy levels, emotional coping skills). The determination of the strength and nature of interactions of these factors, and how they can explain the adaptations seen in response to COVID-19 was not explored in this systematic literature as these are more suited to a meta-analysis methodology. As such, it is recommended that educational policy pay more attention to the emotional-coping mechanisms and problem-focused responses of staff and students as a key to support the ongoing adaptation and learning outcomes.

Despite this, some limitations were identified in this systematic literature review. For example, this review did not compare how digital technology adaptations differentiate between diverse learners. An examination of experiences of learners from different educational levels, age and ethnic background could be conducted in the future. Also, students and staff in certain subjects faced more challenges in online teaching and learning than other subject areas, as not all the pedagogical face to face activities can be transferred to online format. Future research could also examine and compare what digital technologies have been adopted by various disciplines and its related impact. The identified dimensions and sub-factors in this review were based on certain qualitative and quantitative studies that we identified from searching the literature databases, which may skew the results and impact of COVID-19 to specific higher education settings/conditions. Additionally, it was not possible to track adaptations from one wave of lockdown to the next, due to a lack of longitudinal research. This review was also limited to previous empirical studies with sufficient sample sizes to ascertain the factors influencing digital technology adaptation during the COVID-19 pandemic. Therefore, future studies could include different sample sizes and populations. Evaluation of adaptations over the different waves would provide valuable insight into the process of adaptation at a large scale. Finally, papers were not always specific about their level of usage of technologies pre-COVID-19, or always focused on a particular technology and its adaptation in one case. Consequently, targeted studies examining adaptation of impactful technologies from a staff and student perspective across different institutions would enhance the understanding of digital technology adaptation in Higher Education.

## Conclusion

This study reviewed 90 articles to identify the main dimensions and initiatives related to the use of digital technologies during the COVID-19 period. Four dimensions were identified in this work (in relation to first research question): techno-economic; personal and psychological; teaching, learning and assessment; and social. Whilst expected dimensions (such as ease of access, connection between students and staff, self-management, and support) were evident in this systematic review, interesting patterns emerged, such as importance of physical space in online learning, balancing standardization of platforms and applications with need for channels to communicate and engage in the learning community, and flexibility in timetabling (as not related to physical classrooms).

Initiatives (as per the second research question) identified covered national, institutional and individual levels. A clear future practical direction at a national level needs educational policies to recognise online-learning as an effective form of education, and the creation of appropriate national digital learning eco-systems and infrastructure for benefits of scale and consistency. Also, investment in national teaching open educational resources and provision of the framework that aligns these resources with available device and network capabilities is required. At institutional level, policies need to support the creation of pedagogically-underpinned teaching, learning and assessment with the appropriate management support and positive culture to enable staff and students to positively engage with online learning. Additionally, universities need to identify and progress innovation adaptations that have the potential for wider and impactful implementation. Also, mechanisms to support the mental wellbeing issues of staff and students are essential. Finally, universities must gain a deeper individualized perspective on how university infrastructure enables learning for students.

In conclusion, the four dimensions and sub-factors identified in this study overlapped with current models of adoption and adaptation, but this review did not establish the interconnection between these factors. However, the extant models can offer some explanatory and planning insight as universities navigate their way through the ongoing pandemic.

## Supplementary Information

Below is the link to the electronic supplementary material.

Supplementary file1 (DOCX 31 KB)

Supplementary file2 (DOCX 19 KB)
