# Supplementary material for: Digital technology adaptation and initiatives: a systematic review of teaching and learning during COVID-19
Source: J Comput High Educ. 2023 Apr 26:1–22. Online ahead of print. doi: 10.1007/s12528-023-09376-z (PMC10132797; doi:10.1007/s12528-023-09376-z)
Supplement: Supplementary file 1 — Supplementary file1 (DOCX 31 KB) [file 12528_2023_9376_MOESM1_ESM.docx]

**APPENDIX: Table 1.** Brief description of 90 primary studies of the final selection

| **Author year** | **Country** | **Subject/ Discipline** | **Sample** | **Methods** |
| --- | --- | --- | --- | --- |
| Abou-Khalil et al., 2021 | Lebanon and India | N/A | 313 | Survey |
| Ahmed et al, 2021 | Middle Eastern countries (step 1); Egypt (step 2 – focus group) | Medical Education | 79 | Mixed |
| Al-Balas et al., 2020 | Jordan | Clinical Medical education | 588 | Survey |
| Al-Karaki et al., 2021 | United Arab Emirates | N/A | 90 | Survey |
| Al-Rasheed, 2021 | Saudi Arabia | Computer and information Sciences | 68 | Survey |
| Al-Salman and Haider, 2021 | Jordan | Arts & Humanities; Sciences | 4037 | Survey |
| Al-Tarawneh et al., 2021 | Jordan | Engineering | 505 | Survey |
| Almazova et al., 2020 | Russia | General | 87 | Mixed |
| Almohammed et al., 2021 | Saudi Arabia | Pharmacy | 87 | Survey |
| Amir et al., 2020 | Indonesia | Dentistry | 301 | Survey |
| Ariza et al. 2020 | Colombia | Engineering | 287 | Survey |
| Audet et al., 2021 | Canada | N/A | 350 | Survey |
| Ayadat et al., 2021 | Saudi Arabia | UG Science | 200 | Survey |
| Blackley et al., 2021 | Australia | Initial teacher education (ITE) in mathematics, science, and technology (STEM) | 43 | Mixed |
| Bordoloi et al., 2021 | India | Varied (not specified) | 120 | Survey |
| Brooks, 2021 | USA | health services administration program | 12 | Interview |
| Busto et al., 2021 | Italy | Mathematics in Engineering department | 1011 | Survey |
| Calder et al., 2021 | New Zealand | Mathematics | 3 | Interview |
| Casacchia et al., 2021 | Italy | Life, Health and Environmental Science | 97 | Survey |
| Cejas Martinez et al., 2021 | Ecuador | general | 500 | Survey |
| Chen et al., 2021 | USA | Business | 854 | Survey |
| Colfer et al., 2021 | USA | Health Administration | 81 | Survey |
| Costado Dios and Pinero Charlo, 2021 | Spain | Education | 100 | Survey |
| Craig et al., 2020 | USA | Health | 83 | Survey |
| De Ponti et al., 2020 | Italy | Medicine and Surgery | 115 | Survey |
| Eberle & Hobrecht, 2021 | Germany | Chemistry | 15 | Interview |
| El Said, 2021 | Egypt | N/A | 748 | Mixed |
| Elfirdoussi et al., 2020 | Morocco | various | 3268 | Survey |
| Fatani, 2020 | Saudi Arabia | Medicine (pediatrics) | 662 | Survey |
| Frolova et al., 2021 | Russia | Not specified | 158 | Mixed |
| Gautam and Gautam, 2021 | Nepal | Non-numeric; numeric | 158 | Mixed |
| Ghazi-Saidi et al., 2020 | USA | Education | 61 | Mixed |
| Goncalves et al., 2020 | Portugal | General survey including law, social sciences and services; health; Economics, Management and Accounting | 173 | Survey |
| Gradišek & Polak, 2021 | Slovenia | Psychology | 110 | Survey |
| Händel et al., 2020 | Germany | N/A | 1824 | Survey |
| Hattar et al., 2021 | Jordan | Dental | 310 | Survey |
| Hayat et al., 2021 | Iran | Medical sciences | 26 | Interview |
| Hijazi and AlNatour, 2021 | Jordan | EFL | 1200 | Survey |
| Iipinge et al., 2020 | Namibia | Faculties of Management Sciences, Human Sciences, Natural Resources and Spatial Sciences, Computing and Informatics and Engineering | 185 | Survey |
| Johnson et al., 2020 | USA | Not specified | 897 | Survey |
| Kasai et al., 2021 | Japan | Medicine | 43 | Mixed |
| Khairi et al., 2021 | Malaysia | Science | 88 | Survey |
| Khalil et al, 2020 | Saudi Arabia | Medical | 68 | Focus group |
| Klein et al., 2021 | Germany | Physics | 578 | Survey |
| Kuliukas et al., 2021 | Australia | Midwifery | 159 | Mixed |
| Kumar et al., 2021 | India | Graduate Management Studies | 435 | Survey |
| Laher et al., 2021 | South Africa | Psychology | 160 | Survey |
| Lambert and Rennie, 2021 | UK | Engineering Entrepreneurship | 5 | Focus group |
| Langegård et al., 2021 | Sweden | Nursing | 9 | Mixed |
| Lassoued et al., 2020 | Arab World (Algeria, Egypt, Palasine, Iraq) | General | 400 | Survey |
| Liu et al., 2020 | China | Vocational education | 129 | Survey |
| Louis et al., 2021 | USA | Health Administration | 215 | Mixed |
| MacLeod et al., 2021 | USA | Business | 413 | Survey |
| Makgahlela et al., 2021 | South Africa | General | 312 | Survey |
| Malkawi et al., 2020 | United Arab Emirates | N/A | 532 | Survey |
| Martha et al., 2021 | Indonesia | Economics; sociology, politics & humanities; education; engineering; health | 482 | Survey |
| Martinho et al., 2021 | Portugal | General | 65 | Survey |
| Masha'al et al., 2020 | Jordan |  | 355 | Mixed |
| Mok et al., 2021 | China | N/A | 1227 | Survey |
| Montano, 2021 | Phillipines | General | 371 | Survey |
| Morgan et al., 2021 | USA | Pharmacy | 251 | Survey |
| Mouchantaf, 2020 | Lebanon | Languages | 50 | Survey |
| Müller et al., 2021 | Singapore | Not specified | 14 | Interview |
| Nel and Marais, 2021 | South Africa | Teaching | 6 | Mixed |
| Ogbonnaya et al., 2020 | Ghana | N/A | 147 | Survey |
| Oliveira et al., 2021 | Portugal and Brazil | Engineering, applied science, Arts and health Sciences | 30 | Interview |
| Pocsova et al., 2021 | Slovakia | Mathematics | 110 | Survey |
| Puljak et al., 2020 | Croatia | Health Sciences | 2520 | Survey |
| Rasalam and Bandaranaike, 2020 | Australia | Medicine | 66 | Survey |
| Reedy et al., 2021 | Australia | N/A | 1970 | Survey |
| Rizvi and Nabi, 2021 | India | Commerce & Management | 41 | Interview |
| Safonov & Mayakovskaya, 2020 | Russia | N/A | 2560 | Survey |
| Sahbaz, 2020 | Bosnia Herzegova | Turkish (Language) | 40 | Survey |
| Salih and Omar, 2021 | Oman | EFL | 112 | Survey |
| Schlenz et al., 2020 | Germany | Dentistry | 289 | Survey |
| Sebbani et al., 2021 | Morocco | Medical | 111 | Survey |
| Secundo et al., 2021 | Italy | Entrepreneurship | 81 | Mixed |
| Senol et al., 2021 | North Cyprus | Various (health sciences; sports sciences; economics; engineering; dentistry; mathematics; pre-school teaching) | 524 | Survey |
| Shahrvini et al., 2021 | USA | Medicine | 104 | Survey |
| Shawaqfeh et al., 2020 | Saudi Arabia | Pharmacy | 309 | Survey |
| Sim et al., 2021 | Malaysia | Not discipline specific | 156 | Survey |
| Stewart et al., 2021 | USA | general | 511 | Survey |
| Sugino, 2021 | Japan | Political sciences | 59 | Mixed |
| Syauqi et al., 2020 | Indonesia | Mechanical Engineering | 58 | Survey |
| Tavitiyaman et al., 2021 | Hong Kong | Hospitality | 283 | Survey |
| Toader et al., 2021 | Romania | N/A | 1827 | Survey |
| Tuma et al., 2021 | Iraq | Medicine | 717 | Survey |
| Valiyev, 2020 | Azerbaijan | Oil and Gas | 63 | Survey |
| Watermeyer et al., 2020 | UK | Representative of all major disciplines | 1148 | Survey |
| Yu, 2021 | China | Languages | 1152 | Mixed |
