# Supplementary material for: Digital technology adaptation and initiatives: a systematic review of teaching and learning during COVID-19
Source: J Comput High Educ. 2023 Apr 26:1–22. Online ahead of print. doi: 10.1007/s12528-023-09376-z (PMC10132797; doi:10.1007/s12528-023-09376-z)
Supplement: Supplementary file 2 — Supplementary file2 (DOCX 19 KB) [file 12528_2023_9376_MOESM2_ESM.docx]

**APPENDIX: Table 2.** Brief description of dimensions and subfactors

| **Dimensions** | **Sub-Factor (and count)** | **Count** | **Sources** |
| --- | --- | --- | --- |
| Techno-Economic | Internet data | 49 | (Ariza et al. 2020);(Al-Karaki et al., 2021);(Ogbonnaya et al., 2020);(Oliveira et al., 2021);(Toader et al., 2021);(Laher et al., 2021);(Khairi et al., 2021);(Tuma et al., 2021);(Ghazi-Saidi et al., 2020);(Sim et al., 2021);(Bordoloi et al., 2021);(Chen et al., 2021);(Frolova et al., 2021);(Senol et al., 2021);(Syauqi et al., 2020);(Elfirdoussi et al., 2020);(Kasai et al., 2021);(Puljak et al., 2020);(Schlenz et al., 2020);(Lassoued et al., 2020);(Casacchia et al., 2021);(De Ponti et al., 2020);(Iipinge et al., 2020);(Calder et al., 2021);(Sebbani et al., 2021);(Makgahlela et al., 2021);(Masha'al et al., 2020);(Rasalam and Bandaranaike, 2020);(Shahrvini et al., 2021);(Almohammed et al., 2021);(Abou-Khalil et al., 2021);(Malkawi et al., 2020);(Al-Balas et al., 2020);(Al-Rasheed, 2021);(Amir et al., 2020);(Costado Dios and Pinero Charlo, 2021);(Eberle & Hobrecht, 2021);(Fatani, 2020);(Gautam and Gautam, 2021);(Hayat et al., 2021);(Hijazi and AlNatour, 2021);(El Said, 2021) ;(Khalil et al, 2020);(Mouchantaf, 2020);(Rizvi and Nabi, 2021);(Sahbaz, 2020);(Secundo et al., 2021);(Sugino, 2021);(Valiyev, 2020) |
|  | Stability of power/electricity | 9 | (Abou-Khalil et al., 2021);(Gautam and Gautam, 2021);(El Said, 2021) ;(Mok et al., 2021);(Al-Karaki et al., 2021);(Ogbonnaya et al., 2020);(Bordoloi et al., 2021);(Sebbani et al., 2021);(Rasalam and Bandaranaike, 2020) |
|  | Digital devices | 33 | (Abou-Khalil et al., 2021);(Al-Balas et al., 2020);(Al-Rasheed, 2021);(Costado Dios and Pinero Charlo, 2021);(Fatani, 2020);(Gautam and Gautam, 2021);(Händel et al., 2020);(Hijazi and AlNatour, 2021);(Safonov & Mayakovskaya, 2020);(Sugino, 2021);(Valiyev, 2020);(Ogbonnaya et al., 2020);(Khairi et al., 2021);(Tuma et al.,2021);(Ghazi-Saidi et al., 2020);(Sim et al., 2021);(Bordoloi et al., 2021);(Chen et al., 2021);(Frolova et al., 2021);(Senol et al., 2021);(Syauqi et al., 2020);(Watermeyer et al., 2020);(Puljak et al., 2020);(Tavitiyaman et al., 2021);(Goncalves et al., 2020);(Lassoued et al., 2020);(Busto et al., 2021);(Iipinge et al., 2020);(Calder et al., 2021);(Sebbani et al., 2021);(Makgahlela et al., 2021);(Masha'al et al., 2020);(Rasalam and Bandaranaike, 2020) |
|  | Integrated technology/learning platform/Apps | 11 | (Al-Balas et al., 2020);(Fatani, 2020);(Nel and Marais, 2021);(Ghazi-Saidi et al., 2020);(Martha et al., 2021);(Chen et al., 2021);(Elfirdoussi et al., 2020);(Watermeyer et al., 2020);(Tavitiyaman et al., 2021);(Lassoued et al., 2020);(Cejas Martinez et al., 2021); |
| Personal and psychology | Self-motivation and concertation | 41 | (Malkawi et al., 2020);(Al-Salman and Haider, 2021);(Blackley et al., 2021);(Costado Dios and Pinero Charlo, 2021);(Gautam and Gautam, 2021);(Gradišek & Polak, 2021);(Händel et al., 2020);(Hayat et al., 2021);(Hijazi and AlNatour, 2021);(Khalil et al, 2020);(Rizvi and Nabi, 2021);(Secundo et al., 2021);(Sugino, 2021);(Yu, 2021);(Liu et al., 2020);(Mok et al., 2021);(Audet et al., 2021);(Oliveira et al., 2021);(Toader et al., 2021);(Al-Tarawneh et al., 2021);(Tuma et al., 2021);(Ghazi-Saidi et al., 2020);(Sim et al., 2021);(Almazova et al., 2020);(Martha et al., 2021);(Chen et al., 2021);(Johnson et al., 2020);(Müller et al., 2021);(Frolova et al., 2021);(Syauqi et al., 2020);(Watermeyer et al., 2020);(Puljak et al., 2020);(Morgan et al., 2021);(Schlenz et al., 2020);(Hattar et al., 2021);(Tavitiyaman et al., 2021);(Lassoued et al., 2020);(Shawaqfeh et al., 2020);(Colfer et al., 2021);(Louis et al., 2021);(Sebbani et al., 2021); |
|  | Self- independent study skill | 26 | (Malkawi et al., 2020);(Al-Rasheed, 2021);(Amir et al., 2020);(Blackley et al., 2021);(Eberle & Hobrecht, 2021);(Gradišek & Polak, 2021);(Hayat et al., 2021);(Salih and Omar, 2021);(Sugino, 2021);(Ogbonnaya et al., 2020);(Langegård et al., 2021);(Audet et al., 2021);(Oliveira et al., 2021);(Toader et al., 2021);(Ghazi-Saidi et al., 2020);(Sim et al., 2021);(Almazova et al., 2020);(Martha et al., 2021);(Müller et al., 2021);(Senol et al., 2021);(Tavitiyaman et al., 2021);(Goncalves et al., 2020);(Colfer et al., 2021);(Calder et al., 2021);(Craig et al., 2020);(Shahrvini et al., 2021); |
|  | Digital health well-being | 23 | (Malkawi et al., 2020);(Al-Rasheed, 2021);(Costado Dios and Pinero Charlo, 2021);(Khalil et al, 2020);(Montano, 2021);(Rizvi and Nabi, 2021);(Safonov & Mayakovskaya, 2020);(Mok et al., 2021);(Audet et al., 2021);(Toader et al., 2021);(Laher et al., 2021);(Ghazi-Saidi et al., 2020);(Watermeyer et al., 2020);(Morgan et al., 2021);(Tavitiyaman et al., 2021);(Goncalves et al., 2020);(Stewart et al., 2021);(Kumar et al., 2021);(Louis et al., 2021);(Sebbani et al., 2021);(Makgahlela et al., 2021);(Masha'al et al., 2020);(Shahrvini et al., 2021); |
|  | Invasion of privacy (e.g. around proctored exams) | 10 | (Amir et al., 2020);(Gradišek & Polak, 2021);(Sugino, 2021);(Reedy et al., 2021);(Oliveira et al., 2021);(Toader et al., 2021);(Chen et al., 2021);(Kumar et al., 2021);(Casacchia et al., 2021);(Sebbani et al., 2021); |
|  | Staff/student digital skills/literacy | 43 | (Al-Rasheed, 2021);(Fatani, 2020);(Gautam and Gautam, 2021);(Händel et al., 2020);(Hayat et al., 2021);(Hijazi and AlNatour, 2021);(Khalil et al, 2020);(Mouchantaf, 2020);(Rizvi and Nabi, 2021);(Sugino, 2021);(Mok et al., 2021);(Reedy et al., 2021);(Oliveira et al., 2021);(Khairi et al., 2021);(Ghazi-Saidi et al., 2020);(Sim et al., 2021);(Bordoloi et al., 2021);(Almazova et al., 2020);(Martha et al., 2021);(Johnson et al., 2020);(Frolova et al., 2021);(Senol et al., 2021);(Syauqi et al., 2020);(Elfirdoussi et al., 2020);(Watermeyer et al., 2020);(Puljak et al., 2020);(Morgan et al., 2021);(Schlenz et al., 2020);(Tavitiyaman et al., 2021);(Goncalves et al., 2020);(Lassoued et al., 2020);(Busto et al., 2021);(Stewart et al., 2021);(Shawaqfeh et al., 2020);(Cejas Martinez et al., 2021);(Casacchia et al., 2021);(Colfer et al., 2021);(De Ponti et al., 2020);(MacLeod et al., 2021);(Sebbani et al., 2021);(Makgahlela et al., 2021); (Rasalam and Bandaranaike, 2020);(Shahrvini et al., 2021) |
| Teaching, Learning and Assessment | Academic identity (passive dominator to facilitator) | 11 | (Ahmed et al, 2021);(Hijazi and AlNatour, 2021);(Safonov & Mayakovskaya, 2020);(Ghazi-Saidi et al., 2020);(Bordoloi et al., 2021);(Almazova et al., 2020);(Chen et al., 2021);(Müller et al., 2021);(Watermeyer et al., 2020);(Casacchia et al., 2021);(Louis et al., 2021); |
|  | Time implementation (flexibility) | 42 | (Abou-Khalil et al., 2021);(Ahmed et al, 2021);(Al-Balas et al., 2020);(Al-Rasheed, 2021);(Amir et al., 2020);(Ayadat et al., 2021);(Blackley et al., 2021);(Costado Dios and Pinero Charlo, 2021);(Gautam and Gautam, 2021);(Gradišek & Polak, 2021);(Hayat et al., 2021);(Khalil et al, 2020);(Safonov & Mayakovskaya, 2020);(Sahbaz, 2020);(Al-Karaki et al., 2021);(Langegård et al., 2021);(Oliveira et al., 2021);(Toader et al., 2021);(Laher et al., 2021);(Khairi et al., 2021);(Tuma et al., 2021);(Ghazi-Saidi et al., 2020);(Sim et al., 2021);(Almazova et al., 2020);(Martha et al., 2021);(Chen et al., 2021);(Müller et al., 2021);(Senol et al., 2021);(Watermeyer et al., 2020);(Kasai et al., 2021);(Puljak et al., 2020);(Martinho et al., 2021);(Schlenz et al., 2020);(Goncalves et al., 2020);(Casacchia et al., 2021);(Calder et al., 2021);(Klein et al., 2021);(Sebbani et al., 2021);(Makgahlela et al., 2021);(Masha'al et al., 2020);(Shahrvini et al., 2021);(Almohammed et al., 2021); |
|  | Support for staff/students in using technology | 31 | (Al-Balas et al., 2020);(Al-Rasheed, 2021);(Ayadat et al., 2021);(Brooks, 2021);(Eberle & Hobrecht, 2021);(Fatani, 2020);(Hayat et al., 2021);(El Said, 2021); (Lambert and Rennie, 2021);(Mouchantaf, 2020);(Liu et al., 2020);(Ogbonnaya et al., 2020);(Langegård et al., 2021);(Oliveira et al., 2021);(Laher et al., 2021);(Ghazi-Saidi et al., 2020);(Almazova et al., 2020);(Johnson et al., 2020);(Müller et al., 2021);(Elfirdoussi et al., 2020);(Watermeyer et al., 2020);(Schlenz et al., 2020);(Lassoued et al., 2020);(Busto et al., 2021);(Shawaqfeh et al., 2020);(Casacchia et al., 2021);(Colfer et al., 2021);(Sebbani et al., 2021);(Makgahlela et al., 2021);(Masha'al et al., 2020);(Almohammed et al., 2021); |
|  | Acceptance of online/blended teaching (material, approach and technologies) | 64 | (Abou-Khalil et al., 2021);(Al-Rasheed, 2021);(Al-Salman and Haider, 2021);(Brooks, 2021);(Costado Dios and Pinero Charlo, 2021);(Eberle & Hobrecht, 2021);(Fatani, 2020);(Gautam and Gautam, 2021);(Gradišek & Polak, 2021);(Hayat et al., 2021);(Hijazi and AlNatour, 2021);(Khalil et al, 2020);(Lambert and Rennie, 2021);(Mouchantaf, 2020);(Rizvi and Nabi, 2021);(Sahbaz, 2020);(Salih and Omar, 2021);(Secundo et al., 2021);(Sugino, 2021);(Valiyev, 2020);(Yu, 2021);(Ariza et al. 2020);(Mok et al., 2021);(Ogbonnaya et al., 2020);(Langegård et al., 2021);(Toader et al., 2021);(Laher et al., 2021);(Khairi et al., 2021);(Tuma et al., 2021);(Ghazi-Saidi et al., 2020);(Sim et al., 2021);(Bordoloi et al., 2021);(Martha et al., 2021);(Chen et al., 2021);(Johnson et al., 2020);(Müller et al., 2021);(Frolova et al., 2021);(Senol et al., 2021);(Elfirdoussi et al., 2020);(Watermeyer et al., 2020);(Kasai et al., 2021);(Puljak et al., 2020);(Martinho et al., 2021);(Morgan et al., 2021);(Schlenz et al., 2020);(Hattar et al., 2021);(Goncalves et al., 2020);(Lassoued et al., 2020);(Busto et al., 2021);(Stewart et al., 2021);(Shawaqfeh et al., 2020);(Cejas Martinez et al., 2021);(Casacchia et al., 2021);(Colfer et al., 2021);(De Ponti et al., 2020);(Iipinge et al., 2020);(Calder et al., 2021);(Louis et al., 2021);(MacLeod et al., 2021);(Sebbani et al., 2021);(Makgahlela et al., 2021);(Pocsova et al., 2021);(Rasalam and Bandaranaike, 2020);(Shahrvini et al., 2021); |
|  | Practical learning adaptations (Laboratory/placements/clinical) | 12 | (Ahmed et al, 2021);(Amir et al., 2020);(El Said, 2021); (Khalil et al, 2020);(Kuliukas et al., 2021);(Johnson et al., 2020);(Kasai et al., 2021);(Puljak et al., 2020);(Schlenz et al., 2020);(Casacchia et al., 2021);(Masha'al et al., 2020);(Shahrvini et al., 2021); |
|  | Other | 62 | (Abou-Khalil et al., 2021);(Al-Salman and Haider, 2021);(Amir et al., 2020);(Blackley et al., 2021);(Brooks, 2021);(Costado Dios and Pinero Charlo, 2021);(Fatani, 2020);(Händel et al., 2020);(Hayat et al., 2021);(Hijazi and AlNatour, 2021);(El Said, 2021); (Khalil et al, 2020);(Kuliukas et al., 2021);(Lambert and Rennie, 2021);(Mouchantaf, 2020);(Nel and Marais, 2021);(Rizvi and Nabi, 2021);(Sahbaz, 2020);(Salih and Omar, 2021);(Valiyev, 2020);(Yu, 2021);(Al-Karaki et al., 2021);(Oliveira et al., 2021);(Ghazi-Saidi et al., 2020);(Sim et al., 2021);(Bordoloi et al., 2021);(Martha et al., 2021);(Chen et al., 2021);(Johnson et al., 2020);(Müller et al., 2021);(Frolova et al., 2021);(Senol et al., 2021);(Elfirdoussi et al., 2020);(Watermeyer et al., 2020);(Kasai et al., 2021);(Puljak et al., 2020);(Martinho et al., 2021);(Morgan et al., 2021);(Schlenz et al., 2020);(Hattar et al., 2021);(Goncalves et al., 2020);(Lassoued et al., 2020);(Busto et al., 2021);(Stewart et al., 2021);(Shawaqfeh et al., 2020);(Cejas Martinez et al., 2021);(Kumar et al., 2021);(Casacchia et al., 2021);(Colfer et al., 2021);(De Ponti et al., 2020);(Iipinge et al., 2020);(Calder et al., 2021);(Klein et al., 2021);(Louis et al., 2021);(MacLeod et al., 2021);(Sebbani et al., 2021);(Makgahlela et al., 2021);(Masha'al et al., 2020);(Pocsova et al., 2021);(Rasalam and Bandaranaike, 2020);(Shahrvini et al., 2021);(Almohammed et al., 2021); |
| Social considerations | Peer collaboration | 23 | (Amir et al., 2020);(Blackley et al., 2021);(Costado Dios and Pinero Charlo, 2021);(Hijazi and AlNatour, 2021);(Khalil et al, 2020);(Lambert and Rennie, 2021);(Salih and Omar, 2021);(Secundo et al., 2021);(Sugino, 2021);(Ogbonnaya et al., 2020);(Ghazi-Saidi et al., 2020);(Sim et al., 2021);(Chen et al., 2021);(Kasai et al., 2021);(Puljak et al., 2020);(Hattar et al., 2021);(Goncalves et al., 2020);(Casacchia et al., 2021);(Calder et al., 2021);(Craig et al., 2020);(Klein et al., 2021);(Louis et al., 2021);(Almohammed et al., 2021); |
|  | Tutor communication/interaction | 31 | (Abou-Khalil et al., 2021);(Al-Balas et al., 2020);(Brooks, 2021);(Costado Dios and Pinero Charlo, 2021);(Eberle & Hobrecht, 2021);(Gautam and Gautam, 2021);(Hijazi and AlNatour, 2021);(Lambert and Rennie, 2021);(Rizvi and Nabi, 2021);(Sahbaz, 2020);(Salih and Omar, 2021);(Sugino, 2021);(Ariza et al. 2020);(Mok et al., 2021);(Oliveira et al., 2021);(Toader et al., 2021);(Khairi et al., 2021);(Ghazi-Saidi et al., 2020);(Sim et al., 2021);(Almazova et al., 2020);(Müller et al., 2021);(Frolova et al., 2021);(Syauqi et al., 2020);(Watermeyer et al., 2020);(Puljak et al., 2020);(Busto et al., 2021);(Stewart et al., 2021);(Casacchia et al., 2021);(Colfer et al., 2021);(Klein et al., 2021);(Louis et al., 2021); |
|  | Physically space and environment (cost of living, family benefits) | 29 | (Ahmed et al, 2021);(Ayadat et al., 2021);(Brooks, 2021);(Eberle & Hobrecht, 2021);(Gautam and Gautam, 2021);(El Said, 2021); (Khalil et al, 2020);(Kuliukas et al., 2021);(Rizvi and Nabi, 2021);(Safonov & Mayakovskaya, 2020);(Sahbaz, 2020);(Secundo et al., 2021);(Al-Karaki et al., 2021);(Toader et al., 2021);(Laher et al., 2021);(Ghazi-Saidi et al., 2020);(Sim et al., 2021);(Martha et al., 2021);(Müller et al., 2021);(Tavitiyaman et al., 2021);(Goncalves et al., 2020);(Lassoued et al., 2020);(Stewart et al., 2021);(Cejas Martinez et al., 2021);(Casacchia et al., 2021);(Calder et al., 2021);(Louis et al., 2021);(Masha'al et al., 2020);(Shahrvini et al., 2021); |
|  | Learning community (Interaction) | 36 | (Ahmed et al, 2021);(Malkawi et al., 2020);(Al-Balas et al., 2020);(Al-Rasheed, 2021);(Blackley et al., 2021);(Eberle & Hobrecht, 2021);(Fatani, 2020);(Gautam and Gautam, 2021);(Händel et al., 2020);(Hayat et al., 2021);(Hijazi and AlNatour, 2021);(Khalil et al, 2020);(Kuliukas et al., 2021);(Nel and Marais, 2021);(Rizvi and Nabi, 2021);(Sugino, 2021);(Valiyev, 2020);(Ariza et al. 2020);(Mok et al., 2021);(Al-Karaki et al., 2021);(Ogbonnaya et al., 2020);(Langegård et al., 2021);(Oliveira et al., 2021);(Laher et al., 2021);(Ghazi-Saidi et al., 2020);(Almazova et al., 2020);(Chen et al., 2021);(Frolova et al., 2021);(Senol et al., 2021);(Syauqi et al., 2020);(Kasai et al., 2021);(Puljak et al., 2020);(Casacchia et al., 2021);(De Ponti et al., 2020);(Calder et al., 2021);(Craig et al., 2020); |
